# Clarithromycin-Loaded Albumin-Based Nanoparticles for Improved Antibacterial and Anticancer Performance

**DOI:** 10.3390/pharmaceutics17060729

**Published:** 2025-05-31

**Authors:** Walhan Alshaer, Shrouq Alsotari, Nour Aladaileh, Alaa Rifai, Aya Khalaf, Baidaa AlQuaissi, Bushra Sabbah, Hamdi Nsairat, Fadwa Odeh

**Affiliations:** 1 Cell Therapy Center, The University of Jordan, Amman 11942, Jordan; shalsotari@gmail.com (S.A.); noor.omar.aladileh@gmail.com (N.A.); refai.ala278@gmail.com (A.R.); b.alquaissi@ju.edu.jo (B.A.); 2 Department of Chemistry, School of Science, The University of Jordan, Amman 11942, Jordan; 3 Department of Allied Sciences, Faculty of Arts and Sciences, Al-Ahliyya Amman University, Amman 19328, Jordan; a.khaled@ammanu.edu.jo; 4 Department of Biology, School of Science, The University of Jordan, Amman 11942, Jordan; bushrasabbah89@gmail.com; 5 Pharmacological and Diagnostic Research Center, Faculty of Pharmacy, Al-Ahliyya Amman University, Amman 19328, Jordan; h.alnseirat@ammanu.edu.jo

**Keywords:** drug delivery, BSA nanoparticles, clarithromycin, bovine serum albumin, antibacterial

## Abstract

**Background/Objectives:** Clarithromycin (CLA) is a widely used antibiotic effective against a variety of bacterial strains, making it a common treatment for respiratory, skin, and soft tissue infections. Moreover, extensive studies have confirmed the anticancer activity of CLA against different cancers, particularly when combined with conventional therapies. This study investigates the potential anticancer and antibacterial activities of developed CLA-loaded bovine serum albumin nanoparticles (CLA-BSA NPs), designed with optimized physicochemical properties to enhance drug delivery. **Methods**: The CLA-BSA NPs were synthesized using the desolvation method, followed by drug loading. Characterization techniques, including Dynamic Light Scattering (DLS), Fourier-Transform Infrared (FTIR) Spectroscopy, X-Ray Diffraction (XRD), Transmission Electron Microscopy (TEM), and Thermogravimetric Analysis (TGA). **Results**: The results confirmed that CLA interacts with BSA NPs through van der Waals forces. The performance of drug–nanocarrier interaction was further assessed through in vitro drug release studies. The release studies demonstrated that CLA had a robust release profile in reductive media, with a cumulative release of 50.9% in acetate buffer (pH 5.0) supplemented with 10 mM glutathione (GSH). Further biological activity assays were also conducted, including cell viability assays (MTT) and antibacterial activity tests. CLA-BSA NPs demonstrated anticancer activity against the lung cancer (A549) cell line, while showing minimal cytotoxicity on normal human dermal fibroblast (HDF) cells. The antibacterial activity was assessed against *Streptococcus pyogenes*, *Bacillus cereus*, and *Staphylococcus aureus*. Among the tested strains, *Bacillus cereus* exhibited the highest sensitivity, with a minimum inhibitory concentration (MIC) of 0.032 µg/mL, compared to 0.12 µg/mL for *Staphylococcus aureus* and >32 µg/mL for *Streptococcus pyogenes*. **Conclusions**: In conclusion, these findings highlight CLA-BSA NPs as a promising drug delivery system that enhances the anticancer and antibacterial efficacy of CLA.

## 1. Introduction

Macrolides are among the safest broad-spectrum antibiotics used in clinical practice [1]. They are bacteriostatic agents that bind to the 50S ribosomal subunit, thus suppressing the synthesis of proteins important for bacterial growth and replication [2]. They are known for their efficacy in treating a group of Gram-positive and Gram-negative bacteria that invade different tissue types, such as *Streptococcus pneumoniae*, *Streptococcus pyogenes*, *Staphylococcus aureus*, *Haemophilus influenzae*, *Chlamydia trachomatis*, *Treponema pallidum*, and *Mycoplasma pneumoniae*, demonstrating the wide range of applications that the macrolides can target [3,4,5].

The macrolides’ physicochemical properties affect their pharmacokinetics and dynamics [6]. They are hydrophobic in nature, which allows for efficient tissue penetration and cellular accumulation, as evidenced by their large volume of distribution [7]. Alongside high protein binding, the large volume of distribution facilitates a gradual drug release from tissues, resulting in a prolonged half-life [6]. Despite the advantages of having a long half-life and the free movement of the drug among tissues, a reduction in the drug deposition at the site of infection results in therapy failure and the development of bacterial resistance induced by frequent and prolonged antibiotic administration [7]. Moreover, the drugs’ poor hydrophilicity and acid instability limit their absorption into the bloodstream, explaining their poor bioavailability [7,8,9]. These drawbacks have created a need to develop new methods to deliver antibiotics efficiently to the site of action [9].

Since the antibacterial activity of macrolides is concentration-dependent at the site of action, employing drug delivery systems will localize these medical entities in the infected tissues and improve their cellular availability, resulting in rapid clearance of the pathogen and minimizing the possibility of developing resistance [5,7,9]. Drug delivery systems are gaining increasing attention and applications in medical research [5]. These drug delivery systems offer innovative solutions to enhance therapeutic efficacy and pharmacokinetic properties [4,5,9]. Studies have shown enhanced antibacterial activity and cellular bioavailability of antibiotics when they were incorporated into nano-delivery systems, especially for those targeting intracellular bacteria [3,4,7].

Several types of delivery systems have been tested to improve the therapeutic activity of macrolides, such as liposomes [10], PLGA nanoparticles [9], polymeric nanoparticles [11], metal nanoparticles, and albumin-based delivery systems [5,9]. Albumin-based delivery systems are receiving interest among others due to their safety, non-immunogenicity, biodegradability, and availability of multiple binding functionalities, making them the carrier of choice for many drugs. Bovine serum albumin (BSA) consists of 583 amino acids, with a molecular weight of 66.4 kDa and an isoelectric point of 4.7 [12]. As a part of the inflammatory response and tissue injury, overexpression of albumin receptors, specifically GP60, was reported [12]. Exploiting upregulation mechanisms by developing drug-loaded BSA NPs facilitates specific tissue accumulation and offers a sustained drug release within targeted tissues, consequently promoting therapeutic efficacy [9].

Clarithromycin (CLA) is a second-generation, broad-spectrum macrolide derived from erythromycin. It has lower minimum inhibitory concentrations (MICs) and higher acid stability, improving its bioavailability [3,6]. CLA targets various *Streptococcus*, *Staphylococcus*, and *Chlamydia* strains and several atypical pathogens [13]. It is typically used to treat respiratory, skin, and soft tissue infections [3]. Moreover, many studies have reported that CLA, a low-toxicity and low-cost compound, possesses antiproliferative effects against different cancers [14]. Several proposed mechanisms for the anticancer activity of CLA include the direct antiproliferative effect [15], inhibition of autophagy [16], reduction in pro-inflammatory cytokine production [17], and anti-angiogenesis [18]. These multiple mechanisms of action of CLA mark this drug as an attractive candidate for drug repurposing against cancer [14]. For example, a study by Hamada et al. reported that CLA exhibited an antitumor effect against lung cancer as monotherapy [19]. Moreover, a study by Amani et al. showed that the antiproliferative effect of doxorubicin was synergistically enhanced when combined with CLA through the dysregulation of autophagy [16]. 

In this research, we aimed to develop and evaluate the anticancer and antibacterial activity of CLA-loaded BSA nanoparticles (CLA-BSA NPs) with optimized physicochemical specifications against different cancer cell lines and a group of CLA-susceptible bacteria, including *Streptococcus pyogenes*, *Bacillus cereus*, and *Staphylococcus aureus*. We hypothesized that the encapsulation of CLA within BSA NPs would enhance the therapeutic efficacy by improving cellular uptake and providing sustained release. This approach was expected to improve the pharmacokinetics of free CLA, reducing the required dose and thereby minimizing systemic exposure. The focus of this study was to validate this hypothesis through thorough physicochemical characterization, in vitro release studies, and biological evaluation.

## 2. Materials and Methods

### 2.1. Materials

Clarithromycin (CLA) (>99%) was sourced from Dar AlDawa (Amman, Jordan), while BSA (fraction V, ≥98%) and reduced L-glutathione (GSH, ≥98%) were obtained from Sigma-Aldrich (St. Louis, MO, USA), ultrapure water was sourced from Omnia xs systems (Niederahr, Germany), PBS from EuroClone (Milan, Italy), sucrose from Loba chemie (Maharashtra, India), MTT reagent from Bioworld (Dublin, OH, USA), Omnican^®^-100 insulin syringes from Omnican (Melsungen, Germany), dialysis tubing from Medicell Membranes (Cellulose, MW cut-off 12–14 kDa, Size 3 Inf Dia 20/32”-15.9 mm, London, UK), ethanol and acetonitrile from Sharlau chemicals (Barcelona, Spain), potassium dihydrogen phosphate from Sigma Aldrich (St. Louis, MO, USA), triethyl amine from Thermo Scientific™ (Waltham, MA, USA), and orthophosphoric acid from MERCK (Darmstadt, Germany). Dulbecco’s Modified Eagle Medium (DMEM), RPMI-1640, Minimum Essential Medium (MEM), and fetal bovine serum were sourced from Gibco™ (Paisley, UK). Penicillin, streptomycin, L-glutamine, and trypsin were obtained from EuroClone (Milan, Italy).

### 2.2. Preparation of BSA NPs

**Figure 1:**
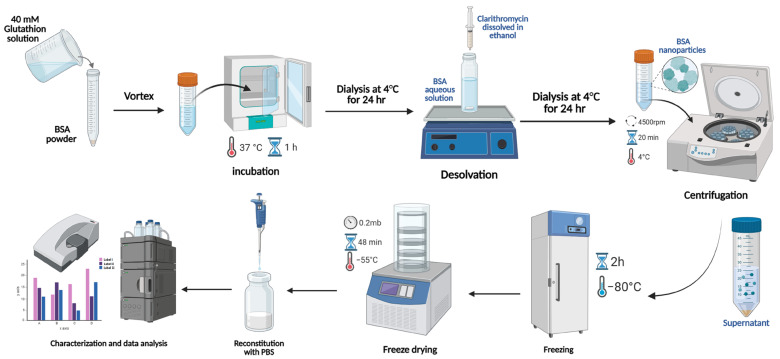
Schematic representation of the synthesis of CLA-loaded BSA NPs using the desolvation method.

In this research, the optimized formula was obtained after testing multiple parameters, including BSA concentrations of 40, 60, 80, and 100 mg/mL; the water-to-ethanol solvent system at ratios of 1:1, 1:2, and 1:4 (*v*/*v*); and CLA drug concentrations of 0, 0.5, 1, and 2 mg. BSA NPs were prepared using a simple desolvation method enhanced by adding a reducing agent, as shown in Figure 1. For loaded BSA NPs, 10 mL of a 40 mM glutathione solution was added to the BSA powder with vortexing until a clear solution was obtained. After that, it was incubated at 37 °C for 1 h. Then, the incubated mixture was dialyzed using deionized water at 4 °C under continuous stirring and left for 24 h; then, the purified samples were collected in glass vials. CLA was solubilized in ethanol and gradually added to the purified samples at a 1 mL/min flow rate with continuous stirring at 37 °C. The mixture was left under stirring until turbidity appeared. The organic solvent and excess drug were removed by dialysis against distilled water at 4 °C overnight (12–14 kDa cut-off). The formed nanoparticles were centrifuged at 4 °C and 4500 rpm for 20 min to eliminate any macromolecules. Finally, the NPs were lyophilized with 5% sucrose using LYOVAPOR/L200 (BUCHI, Essen, Germany). Blank NPs were prepared as loaded NPs but without the drug, and they were used as a control.

### 2.3. Determination of Encapsulation Efficiency (EE) and Drug-Loading (DL) Capacity

The encapsulation efficiency of CLA in CLA-BSA NPs was determined by an indirect method using ultrafiltration (Table 1). After the NPs formed, a predetermined amount of the nanoparticle was added to the ultrafiltration tube (30 kDa cut-off) and then centrifuged at 10,000 rpm for 10 min. The filtrate was placed in a vacuum oven until dry for further analysis.

The drug concentration in the obtained dried filtrate was determined after dilution with the mobile phase (acetonitrile: 50 mM K_2_HPO_4_ buffer +0.8% triethyl amine (pH 3.8) at a 4:6 *v*/*v* ratio). The pH was adjusted with triethylamine and orthophosphoric acid, and it was analyzed using UV-HPLC (Prominence Chromatograph, Shimadzu, Kyoto, Japan) at a 210 nm detection wavelength, 1 mL/min flow rate, and a chromatographic separation through a C-18 column (250 mm × 4.6 mm, 5 µm at 25 °C) (Figure S1) [20].

Encapsulation efficiency (EE) and loading efficiency (LE) were calculated as follows:

EE = Total amount of CLA used in CLA-BSA nanoparticle − Unencapsulated amount of CLA/Total amount of CLA used in CLA-BSA nanoparticle × 100.

LE = weight of CLA encapsulated in CLA-BSA nanoparticle/Total weight of (CLA + BSA) used in formulation × 100.

### 2.4. Size, Polydispersity Index, and Zeta Potential

The nanoparticles’ size, PDI, and zeta potential were measured using Dynamic Light Scattering (ZS Pro, Malvern Zetasizer Nano Instrument, Malvern, UK). For each sample, 20 μL each of the purified CLA-BSA and BSA NPs was diluted in 980 μL of distilled water. The results were recorded in triplicate at 25 °C.

#### 2.4.1. Thermogravimetric Analysis (TGA)

Thermal characteristics were assessed using TGA for BSA, BSA NPs, CLA, CLA-BSA physical mixture, and CLA-BSA NPs. A Mettler Toledo TGA/DSC instrument (Mettler Toledo, Zurich, Switzerland) was used to monitor the TGA curves. Every sample was situated in an alumina crucible and then heated in a range from 30 to 600 °C at a heating rate of 10 °C min^−1^, under a specific continuous nitrogen gas flow (50 mL min^−1^).

#### 2.4.2. Fourier-Transform Infrared (FTIR) Spectroscopy

The infrared spectra of BSA, BSA NPs, CLA, CLA-BSA physical mixture, and CLA-BSA NPs were recorded with an FTIR device (PerkinElmer, Waltham, MA, USA) utilizing an ATR Crystal. The spectra were recorded with a 1 cm^−1^ resolution at a 400–4000 cm^−1^ wavelength scanning region. FTIR spectroscopy analysis was conducted to determine the interaction between CLA and BSA NPs.

#### 2.4.3. X-Ray Diffraction (XRD) Analysis

The XRD patterns of BSA, BSA NPs, CLA, CLA-BSA physical mixture, and CLA-BSA NPs were examined using an X-ray diffractometer (Malvern Panalytical diffractometer, Malvern, UK) using Cu Kα1 radiation as the X-ray source at 40 kV and 7.5 mA. The step size was set at 0.02°/min, and the diffraction diagrams were recorded between 5°, and 50° (2θ).

#### 2.4.4. Transmission Electron Microscopy (TEM)

The morphology of the BSA and CLA-BSA NPs was analyzed using Transmission Electron Microscopy (TEM). A 10 µL sample of each nanoparticle solution was placed onto a copper grid (EMS, Miami, FL, USA) and left to air-dry overnight at room temperature. After that, the samples were stained with uranyl acetate and lead citrate. Then, imaging was conducted using a Versa 3D TEM (FEI, Eindhoven, Netherlands).

#### 2.4.5. Stability Testing

Solubilized CLA-BSA NPs were stored in test tubes screwed with polystyrene caps and subjected to stability testing under three storage temperatures (4 °C, 25 °C, and 37 °C) [21]. The stored samples were then evaluated for particle size, zeta potential, and PDI over a 14-day time interval. At each time point, 10 μL from each sample was diluted up to 1 mL with ultrapure water using Omnia xs systems (Stakpure, Niederahr, Germany) prior to their evaluation. Each run was conducted in triplicate.

### 2.5. In Vitro Release

In vitro cumulative release (CR) of CLA from CLA-BSA NPs was carried out at 37 °C in different conditions: in PBS (pH 7.4) and in 0.1 M sodium acetate buffer (pH 5.0), with and without a reducing agent (10 mM GSH). All release media contained 0.5% SLS (*w*/*v*) to ensure sink conditions.

The freeze-dried NPs were suspended in Eppendorf tubes containing 1 mL of medium to give a final drug concentration of 44 µg/mL. The incubation and shaking were carried out at 37 °C and 100 rpm, respectively, for 3 days. At regular time points, each tube was removed and centrifuged at 15,000 rpm for 20 min. Then, 40 µL of supernatant was withdrawn and replaced with an equivalent volume of fresh medium. The pellets were resuspended by vortexing and sonication for a few minutes. Furthermore, HPLC was used to quantify the drug in the supernatant. Each experiment was conducted in triplicate [22]. The percentage of CR was calculated as follows:

(1) $$\%CR\;of\;CLA=\frac{[CLA]t}{[CLA]total}\times 100\%$$

where [CLA]t is the concentration of released CLA at time t, and [CLA]total is the total concentration of loaded CLA in the NPs.

### 2.6. Anticancer Activity: Cell Viability Assay (MTT)

Cell lines: Human dermal fibroblasts (HDFs; ATCC number: PCS-201-010), triple-negative breast cancer (MDA-MB-231; ATCC number: HTB-131), progesterone- and estrogen-positive breast cancer (MCF-7; ATCC number: HTB-22), and adenocarcinoma human alveolar basal epithelial cells (A549; ATCC number: CCL-185) were obtained from the Global Bioresource Center (ATCC).

MCF7, MDA-MB-231, A549, and HDF cell lines were seeded at 9000 cells/well in 96-well plates. After 24 h, the cells were treated with serial concentrations of free CLA and CLA-BSA NPs, ranging from 0.1 to 100 μM. After 72 h of treatment, the cells were subjected to 10 μL of 3-(4,5-dimethyl-2-thiazolyl)-2,5-diphenyltetrazolium bromide (MTT) solution (Bioworld, Visalia, CA, USA). After 4 h of incubation at 37 °C, the media were aspirated, and the reduced formazan dye was solubilized by adding 50 μL of dimethyl sulfoxide (DMSO). The absorbance was measured at a wavelength of 570 nm using a Glomax microplate reader (Promega, Madison, WI, USA).

### 2.7. Antibacterial Activity Assay

#### 2.7.1. Bacterial Strains and Growth Conditions

Three bacterial strains, *Bacillus cereus* (ATCC 63301), *Staphylococcus aureus* (ATCC 6538), and *Streptococcus pyogenes* (ATCC 19615), were investigated for testing the CLA-BSA nanoparticles’ antibacterial efficiency. First, the strains were inoculated in Mueller–Hinton broth and were incubated in an orbital shaker with a shaking speed of 200 rpm/min at 37 ± 1 °C overnight. The inoculated strains were then diluted in broth to be adjusted to a 0.5 McFarland standard of turbidity and an optical density within 0.08–0.13 when measured at 630 nm wavelength with a UV spectrophotometer.

#### 2.7.2. Antibacterial Activity: Disk Diffusion Test

The nanoparticles’ antibacterial activity was evaluated by performing a disk diffusion test [23]. Briefly, Mueller–Hinton agar was prepared and autoclaved prior to its distribution over sterile Petri dishes, with about 15 mL per plate. The plates were left on a flat surface to solidify at room temperature for 15 min. A sterile swab was then used to take a swab from pre-prepared inoculums of the bacterial strains (*Bacillus cereus* (ATCC 63301) and *Staphylococcus aureus* (ATCC 6538)), and they were spread evenly over the surface of the agar medium in three directions by rotating the plates approximately 60° for even distribution, each in triplicate. Following inoculation, the agar surface was allowed to dry. Each inoculated plate was divided into 4 quadrates, each of which was treated with one of the following treatments: ampicillin (AM10) as a positive control, free CLA, unloaded BSA nanoparticles, or CLA-BSA nanoparticles. Antimicrobial disks of ampicillin were placed over agar using sterile forceps and slightly pressed down to ensure their contact with the agar. After cutting holes, 50 µL of free CLA, unloaded BSA, and CLA-BSA NPs (at a drug concentration of 8 µg/mL) was loaded onto the agar. The plates were then incubated at 37 °C for 24 h, and the zone of inhibition was measured using a ruler [23].

#### 2.7.3. Antibacterial Activity: Minimum Inhibitory Concentrations (MICs)

The nanoparticles’ antibacterial activity was evaluated through determination of their minimum inhibitory concentrations (MICs) using a turbidimetric method provided by the Clinical and Laboratory Standards Institute (CLSI) [24]. First, stock solutions of the drug and nanoparticles (loaded or unloaded) were prepared by dissolving a sufficient amount of each in methanol or sodium acetate buffer, respectively. Nine serial dilutions were performed in broth (0.125–64 µg/mL) from each preparation of free CLA, CLA-BSA NPs, and BSA NPs, and then they were distributed in triplicate into 96-well plates. Finally, the previously prepared bacterial suspensions were diluted at a 1/100 ratio, and a 100 µL sample was taken and added to the wells loaded with these treatments, achieving a final bacterial concentration of 10^5^ cells/mL and a final drug concentration range of 0.0625–32 µg/mL. Broth media and sodium acetate were used as negative controls, and CLA was used as a positive control. All plates were incubated overnight in an orbital shaker (100 rpm/min) at 37 °C. The MIC was determined as the minimum concentration at which there was no visible change in the turbidity of the medium [25].

## 3. Results and Discussion

### 3.1. Preparation of BSA NPs

**Figure 2:**
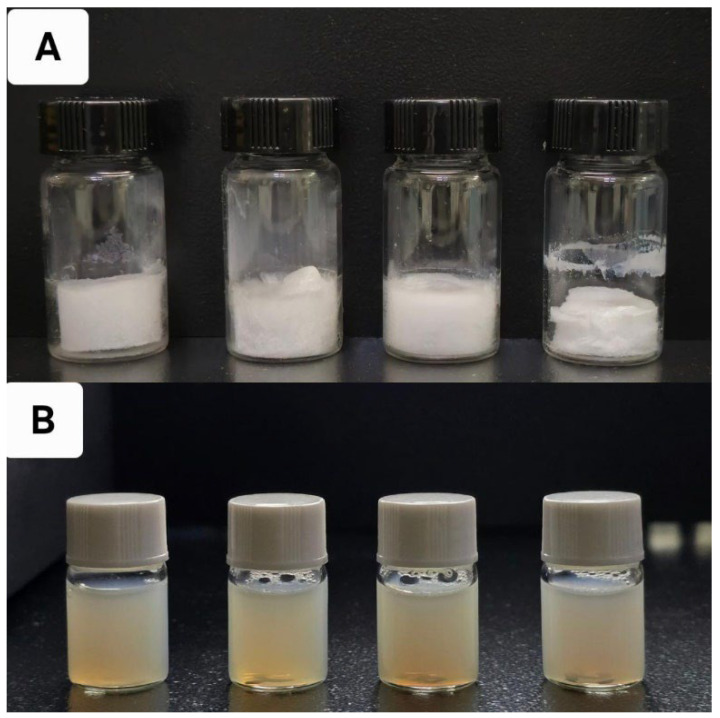
The physical appearance of (**A**) lyophilized BSA NPs containing 0, 0.5, 1, and 2 mg of CLA, from left to right, and (**B**) the same samples after reconstitution with PBS.

The nanoparticles were prepared using a simple desolvation method (Figure 1 and Figure 2), which is commonly used for synthesizing albumin-based nanoparticles [26,27,28,29]. Figure 2 shows the appearance of CLA-BSA NPs after freeze-drying (Figure 2A) and after reconstitution (Figure 2B). The freeze-dried CLA-BSA NPs exhibited a fluffy and uniform appearance. Moreover, upon reconstitution, the colloidal appearance of the CLA-BSA NPs indicated a stable NP suspension, suggesting preserved integrity of the NPs. Importantly, a BSA concentration of 100 mg/mL was also tested, but it was excluded due to the formation of a viscous, gel-like material upon incubation with the reducing agent.

**Figure 3:**
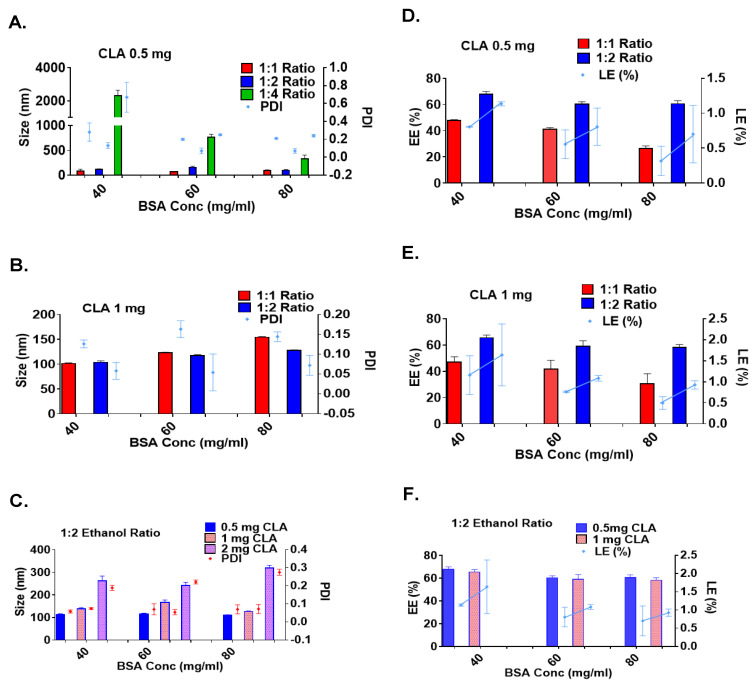
Size, polydispersity index, and zeta potential: (**A**) Particle size and PDI at 0.5 mg/mL CLA-BSA NPs, and (**B**) at 1 mg/mL, across varying ethanol volumes. (**C**) The effects of CLA and BSA concentration on particle size and PDI of BSA NPs at a 1:2 water/EtOH ratio. (**D**,**E**) Encapsulation efficiency (EE%) and loading efficiency (LE%) at different organic solvent volumes for 0.5 and 1 mg/mL CLA concentrations. (**F**) Effects of CLA and BSA concentration on encapsulation efficiency EE% and loading efficiency LE% of BSA NPs at a 1:2 water/EtOH ratio.

The results in Figure 3 and Table 1 show that, at each BSA concentration, the particle size increased as the ethanol-to-water ratio increased. This trend can be attributed to a significant reduction in the dielectric constant of the overall solution, leading to enhanced phase separation and particle aggregation. The dielectric constant of the solution is influenced by its composition and the proportion of each solvent used. Studies have shown that even small volumes of ethanol can precipitate proteins, with turbidity appearing quickly due to the lower dielectric constant of ethanol (24.5) compared to water (88). Increasing the ethanol percentage to the critical radius leads to higher particle density without affecting the particle size. However, exceeding the critical radius produces particle aggregation and phase separation, which explains the larger particle size, increased turbidity, high polydispersity, and higher precipitation yield observed at a 1:4 H_2_O/EtOH ratio [28,29,30].

However, when examining the effect of BSA concentration on a fixed water-to-ethanol ratio, the particle size decreased as the BSA concentration increased, contradicting the findings reported in previous studies [29,31]. Several studies have shown that BSA NPs exhibit a concentration-dependent assembly [30,32]. Accordingly, as the BSA concentration increases, more protein molecules are available for nanoparticle formation, and the shorter distance between albumin molecules allows for protein–protein interactions. This leads to faster nanoparticle self-assembly and more nucleation sites, resulting in a larger number of smaller albumin particles with a narrow particle size distribution [33]. However, exceeding the critical radius of BSA-NPs encourages the nucleation of smaller albumin particles on the surfaces of the larger ones, which is induced by surface interactions and surface free energy; thus, larger particles with a broader particle size distribution, followed by aggregation and particle precipitation, will be obtained [33]. The results obtained in this research were consistent with those of Rahimnejad et al. [34], since the centrifugation process after particle formation was used to purify the formulae from the large and aggregated particles, resulting in a small particle size and narrow PDI at high BSA concentrations.

Based on the results presented in Table 1 and Figure 3, BSA concentrations of 40, 60, and 80 mg/mL were used at 1:1 and 1:2 water-to-ethanol ratios for further testing with 1 mg/mL CLA. Studies have shown that the characteristics of produced albumin-based delivery systems vary in response to drug concentration, depending on the drug’s physicochemical properties and the nanoparticles’ preparation conditions [29,33,35]. In the case of CLA, the size of the BSA-NPs increased as the drug-to-BSA ratio increased, with minor changes in the particle size distribution. These findings are consistent with previous studies on 5-fluorouracil-, piceatannol-, and sulfasalazine-loaded albumin delivery systems [29,36,37,38]. These studies discussed the effects of different drug concentrations on the BSA NPs’ properties, which showed that the higher the drug concentrations, the larger the nanoparticles. CLA exhibits better drug–protein interactions, resulting in protein conformational changes and particle size variations. However, exceeding the nanoparticles’ drug-loading capacity resulted in large particle formation and significant loss of drug and BSA molecules by precipitation, resulting in reduced encapsulation efficiency (EE%) and loading efficiency (LE%). Based on the findings shown in Figure 3, the formula prepared using 40 mM glutathione, a 40 mg/mL BSA concentration, and 1 mg/mL CLA at a 1:2 water-to-ethanol ratio was the selected formula for the subsequent analysis.

**Figure 4:**
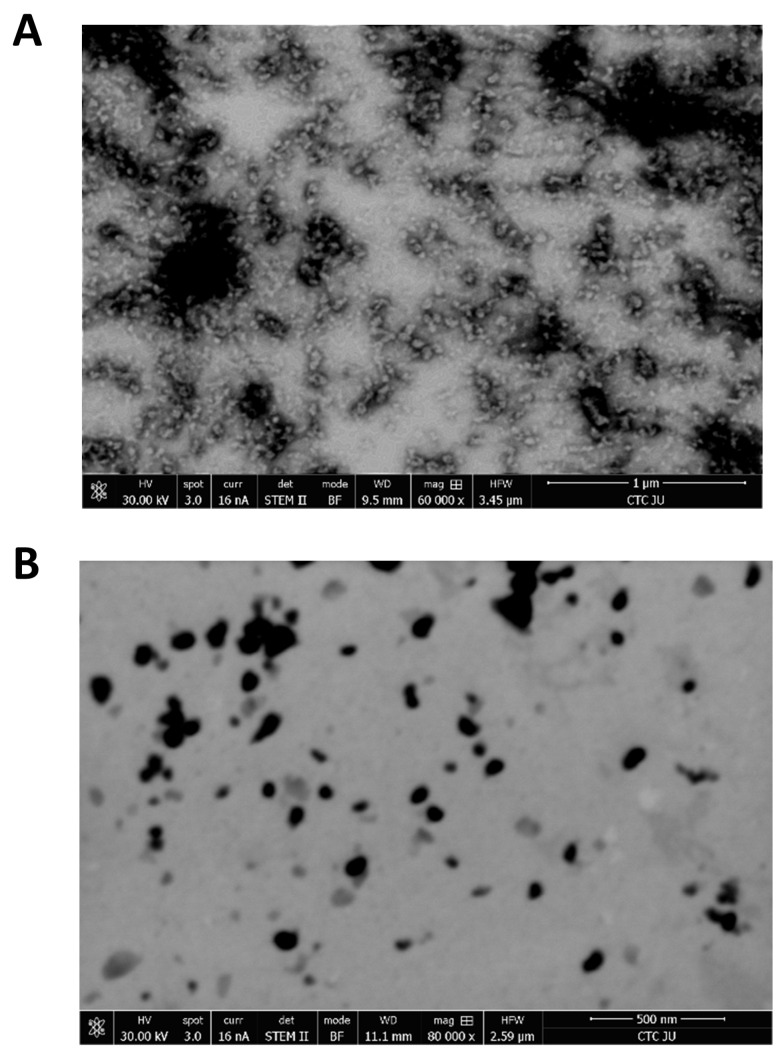
TEM images of (**A**) BSA NPs and (**B**) CLA-BSA NPs.

TEM analysis was performed to analyze the size and morphology of the BSA and CLA-BSA NPs. Figure 4 shows that BSA NPs exhibit a smooth, roughly spherical shape with an empty core. In contrast, the CLA-BSA NPs appear slightly larger, with subtle surface texture changes, indicating successful drug encapsulation. Differences in electron density, morphology, slight aggregation, and the presence of dark-core particles further confirm the incorporation of CLA within the nanoparticles. These findings highlight the potential of BSA NPs as effective drug carriers, enhancing the solubility and bioavailability of CLA [39].

### 3.2. Evaluation of the Prepared Nanoparticles

#### 3.2.1. Thermogravimetric Analysis (TGA)

**Figure 5:**
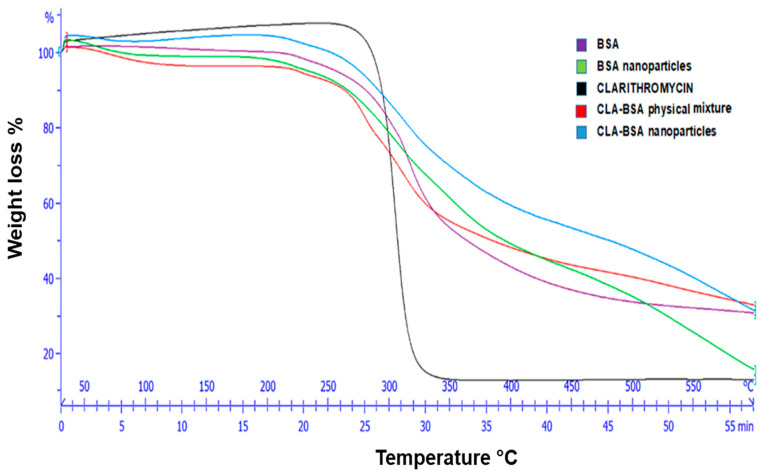
TGA graph of CLA, BSA, BSA NPs, CLA-BSA physical mixture, and CLA-BSA NPs.

Figure 5 presents the TGA thermograms of pure CLA, pure BSA, blank BSA NPs, the CLA–BSA physical mixture, and CLA-BSA NPs. For pure CLA, a major thermal paradigm shift and intense mass loss for CLA were observed at the range 250–300 °C and above 300 °C, respectively [40]. This indicates that CLA’s drug degradation is above its melting point, although CLA shows decomposition and unstable behavior at elevated temperatures [41,42]. For BSA and blank BSA NPs, the results show that the major degradation for BSA occurs between 250 and 350 °C, which can be attributed to protein denaturation and peptide bond breakdown. Similarly, blank BSA NPs have the same trend as the BSA curve, with a slight shift in thermal transitions related to structural modifications resulting from nanoparticle formulation. The CLA–BSA physical mixture showed overlapping degradation profiles of the CLA and BSA curves, suggesting no significant thermal interactions. The CLA-loaded BSA NPs showed improved thermal stability, as evidenced by their late degradation compared to pure CLA. This shift suggests that encapsulation within the BSA matrix protects CLA from early thermal degradation. Moreover, the thermal degradation profile of the loaded NPs aligns more with the BSA profile, further confirming the successful encapsulation of CLA within the protein matrix [43,44].

#### 3.2.2. Fourier-Transform Infrared (FTIR) Spectroscopy

**Figure 6:**
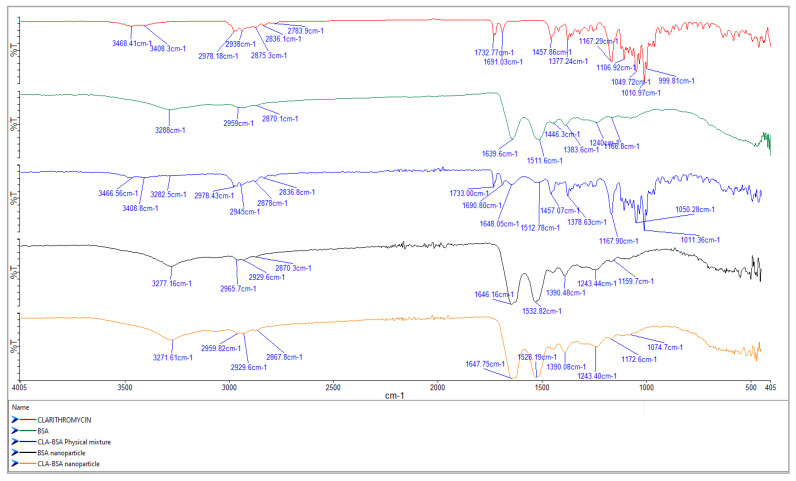
FTIR spectra of CLA, BSA, BSA NPs, CLA-BSA physical mixture, and CLA-BSA NPs.

Analysis of the molecular interactions of the main functional groups for CLA, BSA, BSA NPs, CLA-BSA physical mixture, and CLA-BSA NPs was performed using FTIR spectroscopy, and the results are presented in Figure 6.

The primary characteristic bands of BSA were found during the analysis at 3279.43 cm^−1^ (amide A, concerning N-H stretching), 2954.9 cm^−1^ (amide B, which is the N-H stretching of NH_3_^+^ free ions), 1639.28 cm^−1^ (amide I, C=O rotational contour), 1512.94 cm^−1^ (amide II, due to C-N vibration stretching and N-H bending torsional vibration), 1442.7 cm^−1^ (bending group of CH_2_), and 1384.1 cm^−1^ (amide III, associated with CN stretching and N-H in-plane folding) [39,45,46]. For BSA NPs, the broadening or shift of the BSA peaks suggests a change in the protein structure due to the nanoparticle formation. The raw spectrum and peaks produced by the CLA are usually correlated with O-H stretching (3471 cm^−1^), C-H stretching (2977 cm^−1^), and lactone ring C=O stretching (1730.5 cm^−1^) [40].

The physical mixture spectrum demonstrates no change in the peaks of BSA and CLA, indicating no significant molecular interactions, and the simple mixing would not result in new chemical bonds or structural changes. In contrast, the BSA NPs loaded with CLA showed slight shifts and changes in peak intensity in the amide and lactone carbonyl regions (~1650 cm^−1^ and ~1730 cm^−1^), suggesting molecular interactions between BSA and CLA. These interactions could be hydrogen bonding or electrostatic interactions, confirming the successful encapsulation of CLA into the BSA NPs.

#### 3.2.3. X-Ray Diffraction (XRD) Analysis

**Figure 7:**
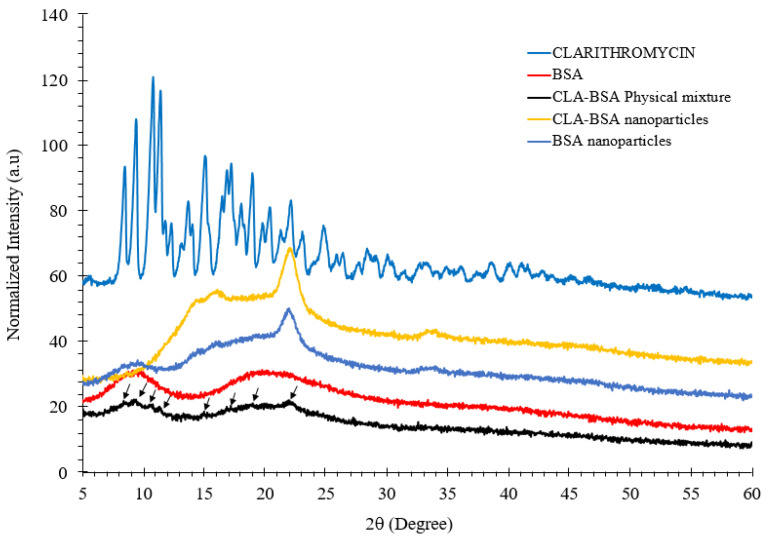
XRD spectra of CLA, BSA, BSA NPs, CLA-BSA physical mixture, and CLA-BSA NPs. The black arrows indicate the characteristic crystalline peaks of CLA retained in the CLA-BSA physical mixture.

Figure 7 shows the XRD patterns of pure CLA, BSA, BSA NPs, CLA-BSA physical mixture, and CLA-BSA NPs. The curve corresponding to pure CLA displays prominent, sharp, and intense peaks within the 2θ range of 9° to 25°, indicating a high degree of crystallinity with large crystallite size [47].

The XRD pattern of pure BSA displays a completely amorphous structure, characterized by a broad, featureless profile without sharp peaks, reflecting the disordered arrangement of its protein chains. The main peak at 2θ of 23° for both CLA-BSA and BSA NPs is sharp, while that for BSA is broad. Sharp peaks indicate the presence of a crystalline structure, while broad peaks indicate an amorphous structure. This observation implies that synthesizing BSA NPs results in partial crystallization of the protein matrix, and a similar effect occurs in the CLA-BSA NPs [48,49]. In addition, CLA-BSA NPs exhibit broader peaks compared to pure CLA, indicating a partial transformation from a highly crystalline CLA to a more amorphous state, suggesting that clarithromycin is encapsulated within the BSA matrix, where the overall crystallinity of the drug is reduced [50]. This change is likely due to drug entrapment, molecular dispersion, and interaction with the nanoparticle matrix, leading to reduced crystallinity, exhibiting a similar amorphous profile, but lacking the characteristic crystalline peaks associated with CLA, which is beneficial for controlled drug release and improved dissolution properties [49,51]. Finally, the CLA-BSA physical mixture retained CLA’s crystallinity, suggesting no strong interactions between the CLA and BSA.

#### 3.2.4. Stability Testing

**Figure 8:**
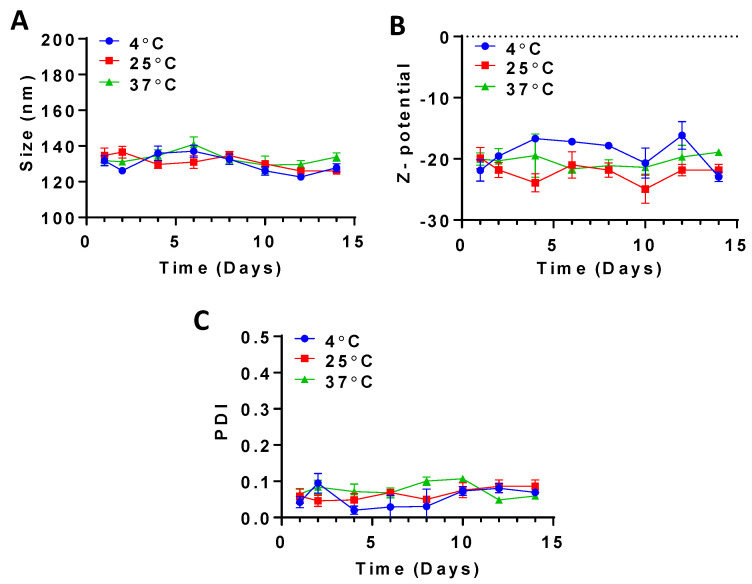
Stability testing of CLA-BSA NPs over 14 days, measuring (**A**) size, (**B**) Z-potential, and (**C**) PDI.

Over 14 days of stability testing, the average size of the CLA-BSA NPs exhibited a fluctuating pattern within a constant range, a consistent and narrow PDI over all storage temperatures (4 °C, 25 °C, and 37 °C), and the least size fluctuation was observed at 37 °C, suggesting higher stability (Figure 8). The average size varied slightly, in the range of 121 to 143 nm; this minimal variation could be attributed to the alteration in water content within or at the surface of the nanoparticles, resulting in a slight swelling or shrinkage and a subsequent change in hydrodynamic diameter over a long duration, and this was found to be significant at higher temperatures. Similar findings have also been reported for other BSA NPs [52,53,54,55]. Furthermore, a limited variation was noted for the nanoparticles’ surface charge, between −16 and −23 mV.

### 3.3. In Vitro Release

**Figure 9:**
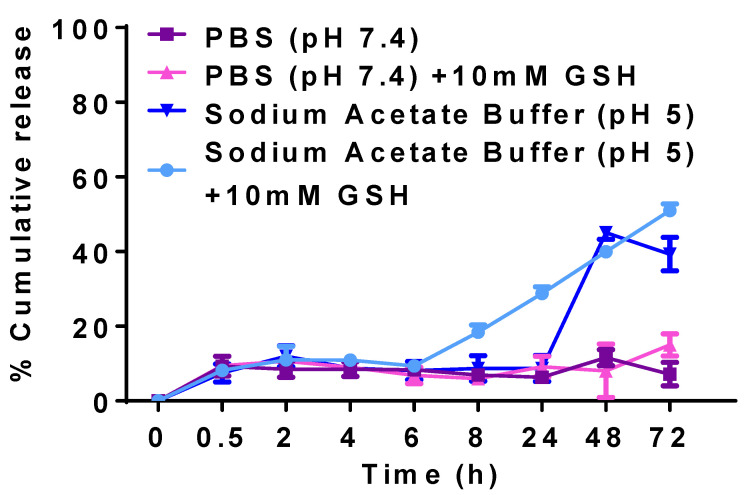
Cumulative release curves of CLA-BSA NPs under different conditions: CLA release by using PBS at pH 7.4, CLA release by using PBS at pH 7.4 + 10 mM GSH, CLA release by using acetate buffer at pH 5.0, and CLA release by using acetate buffer at pH 5.0 + 10 mM GSH.

Figure 9 presents the cumulative release of CLA from CLA-BSA NPs under different conditions. The results demonstrate enhanced release of CLA in reductive conditions compared to non-reductive conditions. Notably, there was no initial burst release, indicating a uniform encapsulation. In the case of PBS and sodium acetate buffer, where the media are free from reducing agents (GSH), CLA-BSA NPs exhibited a cumulative release of 6.36% and 8.69% after 8 h, respectively. After 72 h, the cumulative release reached 5.61% in PBS and 39.32% in sodium acetate buffer. The stability of the NPs explains their observed behavior under physiological conditions. This release may be due to surface-associated CLA in the nanoparticles (adsorption mechanism) [52,56].

For mimicking the reductive microenvironments of cytosol and lysosome, 10 mM GSH was added to PBS and sodium acetate buffer. In GSH-supplemented PBS, ~6% of CLA was released after 6 h, increasing to 14% after 72 h. Compared with PBS, only 9.36% of the drug was released in acidic buffer, reaching 50.9% after 72 h. Here, CLA’s slower and sustained release behavior may have been due to the slow diffusion of entrapped drug from the NPs. This mode of release is beneficial at tumor sites and inside the cells, where the pH is lower, and the environment is reductive compared to the normal environment. As shown in Figure 9, different release mechanisms were observed: adsorption onto the surface of NPs, and entrapment within the matrix [53]. The presence of GSH, a reducing agent, significantly accelerated the release. Therefore, albumin could be considered a bioreducible polymer that can undergo thiol–disulfide exchange under reductive conditions [57].

### 3.4. Cell Viability Assay (MTT)

**Figure 10:**
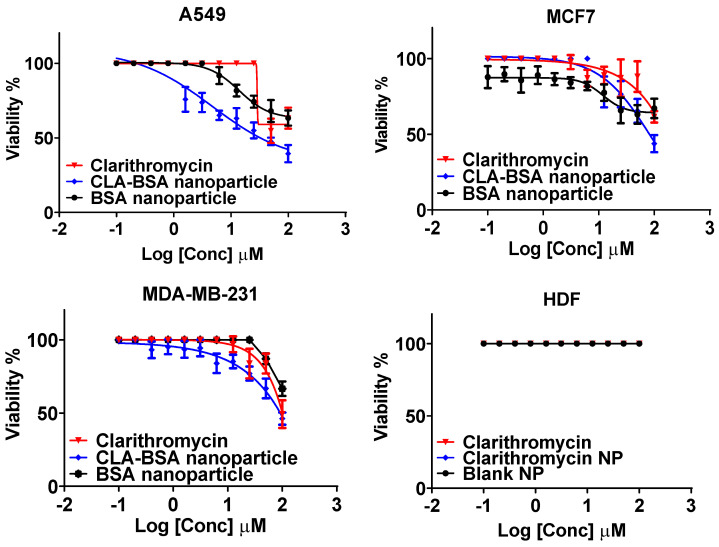
The anticancer activity of CLA, BSA NPs, and CLA-BSA NPs against lung cancer (A549), breast cancer (MCF7, MDA-MB-231), and human dermal fibroblasts (HDFs).

Figure 10 and Table 2 show the anticancer activity and the IC_50_ values of free CLA, BSA NPs, and CLA-BSA NPs against breast cancer cell lines (MCF7, MDA-MB-231) and a lung cancer cell line (A549), as well as their cytotoxicity towards normal human dermal fibroblast cells (HDFs). Generally, our results showed higher antiproliferative activity for the CAL-BSA-treated cancer cell lines (A549, MCF7, and MDA-MB-231) than for the normal cell line (HDFs), indicating higher selectivity against cancer cells. This important finding may be due to improved drug stability, uptake, and release kinetics [58].

CLA (free and loaded) showed reasonable anticancer activity against A549 cancer cells, with IC_50_ values of 66.1 ± 11.3 μM for free CLA and 47.5 ± 9.6 μM for loaded CLA. These findings are consistent with those of a study by Wada et al. testing the anticancer activity of clarithromycin on human lung adenocarcinoma cells [59]. The research has revealed that clarithromycin does not affect the cell proliferation activity but decreases the invasiveness of A549 cells in a dose-dependent manner by suppressing the expression of specific inflammatory mediators, such as thymidine phosphorylase (TP), as well as α_2_ and β_2_ integrins. Moreover, several clinical studies have shown that long-term treatment with clarithromycin in patients with non-small-cell lung cancer (NSCLC) prolongs survival rates by minimizing the production level of serum IL-6, which is directly correlated with cachectic status [60,61]. However, it does not have any effect on disease progression.

On the other hand, results have shown insignificant anticancer activity against MDA-231 and MCF-7 cell lines, with IC_50_ > 100 µM. A study conducted by Amani et al. showed that more than 90% viability of MCF-7 cells was observed after 48 h of incubation with clarithromycin concentrations ranging from 5 to 100 µM, and this matches our finding that monotherapy with clarithromycin does not exert anticancer activity against MCF-7 cells [16]. The same results were found for MDA-MB-231 cells by Komatsu et al., who found that monotherapy with clarithromycin cannot stand alone against triple-negative breast cancer [62]. However, it enhances anticancer agents’ cytotoxicity when administered in combination. Incorporating clarithromycin into BSA NPs did not significantly improve the anticancer activity against all tested cell lines. Regarding safety and biocompatibility, our results are consistent with those of previous reports showing that both clarithromycin and albumin-based delivery systems are safe and nontoxic agents [1,12].

### 3.5. Antibacterial Activity 

**Figure 11:**
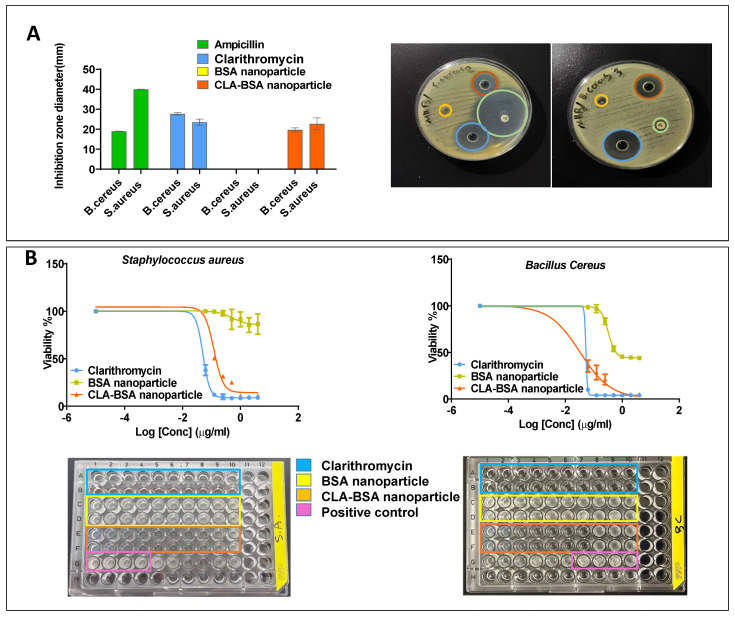
Antibacterial activity of CLA-BSA NPs: (**A**) Well diffusion of two bacterial strains (*Bacillus cereus*, *Staphylococcus aureus*), treated with ampicillin, free CLA, BSA NPs, and CLA-BSA NPs. (**B**) MIC of two bacterial strains (*Bacillus cereus*, *Staphylococcus aureus*), treated with ampicillin, free CLA, BSA NPs, and CLA-BSA NPs.

The antibacterial efficacy of CLA-BSA NPs was evaluated against *Staphylococcus aureus*, *Bacillus cereus*, and *Streptococcus pyogenes*. The findings showed a good inhibitory effect against *Staphylococcus aureus* and *Bacillus cereus,* with less activity noticed for *Streptococcus pyogenes* at the highest tested concentrations. Moreover, the results strongly suggest the efficacy of the CLA-BSA NPs formulation, with marginal activity differences compared to free CLA. The disk diffusion assay showed a slightly higher susceptibility of *Bacillus cereus* strains to CLA-BSA NPs than that of *Staphylococcus aureus* strains, with average inhibitory zone diameters of 22.7 ± 0.25 mm and 19.7 ± 0.057 mm, respectively. This activity was comparable to free CLA treatment, with an inhibitory zone diameter of 27.7 ± 0.058 mm and 23.5 ± 0.11 mm for *Bacillus cereus* and *Staphylococcus aureus*, respectively. The blank BSA NPs showed no antibacterial activity, with no inhibitory zone, as illustrated in Figure 11A.

Figure 11B and Table 3 illustrate comparable results of the MIC values for *Bacillus cereus* and *Staphylococcus aureus. Bacillus cereus* was the most susceptible strain among the treated strains, with an MIC of about 0.053 µg/mL and 0.032 µg/mL for free CLA and CLA-BSA NPs, respectively. Moreover, the MIC of the treated *Staphylococcus aureus* was found to be 0.05 µg/mL and 0.12 µg/mL, respectively.

The results showed insignificant antibacterial improvement of the CLA-BSA NPs compared to free CLA, which can be explained by multiple phenomena. Firstly, albumin receptors are absent on bacterial surfaces. This research’s rationale mainly comes from the finding that albumin-based nanoparticles are efficient carriers for antibiotics targeting intracellular bacteria [3,4,9]. The inflammation that occurs because of bacterial invasion stimulates the overexpression of albumin receptors on the surfaces of the infected tissue [14], resulting in better cellular uptake and accumulation by the invaded cells but not by bacteria [9]. Thus, incorporating clarithromycin inside the albumin delivery system will localize it inside the infected cells, resulting in an elevation of drug deposition at the site of infection where the bacteria are found [3,4,7,63]. Secondly, the drug release from albumin-based systems is enzyme-mediated, so it is expected to achieve rapid pathogen clearance in inflamed tissues, since they offer high enzymatic activity, resulting in rapid drug release and deposition. [5,7,9]. In this model, the prepared nanoparticles were directly added to the bacterial culture without having invaded the cells with the bacteria of interest. Additionally, the culture media used here lacked enzymes necessary for drug release, so the activity was release-dependent, since the antimicrobial activity is concentration-dependent [9]. Thus, there is a need for more relevant assays that evaluate the antibacterial activity of antibiotic-loaded BSA NPs against intracellular bacteria.

## 4. Conclusions

This study successfully synthesized clarithromycin-loaded bovine serum albumin nanoparticles (CLA-BSA NPs) using the desolvation method and characterized them by DLS, XRD, FTIR, TGA, and TEM. The results confirmed uniform size (ranging between 130 and 140 nm), high encapsulation efficiency (65.9%), and enhanced release (50.9%) in reductive acetate buffer containing GSH. The CLA-BSA NPs showed superior antibacterial activity, particularly against *Bacillus cereus*, with a minimum inhibitory concentration (MIC) of 0.032 µg/mL, compared to 0.053 µg/mL for free CLA. Furthermore, CLA-BSA NPs demonstrated promising anticancer effects against A549 lung cancer cells, with minimal effect against normal human dermal fibroblast cells (HDFs). To the best of our knowledge, CLA-BSA NPs were used as nanocarriers for the first time to enhance CLA delivery in different types of cells. The findings of this study confirm the potential of CLA-BSA NPs to improve the pharmacological efficacy and targeted delivery of CLA for both anticancer and antibacterial applications.

## Figures and Tables

**Table 1:** Summary of particle size, polydispersity index (PDI), zeta potential, encapsulation efficiency (EE%), and loading efficiency (LE%) for BSA and CLA-BSA NPs before and after lyophilization.

	Before Lyophilization		After Lyophilization	
Nanoparticle Type	BSA NPs	CLA-BSA NPs	BSA NPs	CLA-BSA NPs
Water/Et Ratio	1:2	1:2	1:2	1:2
BSA Concentration (mg/mL)	40	40	40	40
Drug Amount (mg/mL)	-	1	-	1
Size (nm)	99.4 ± 0.68	140.5 ± 2.97	87.9 ± 2.27	129.4 ± 2.25
PDI	0.083 ± 0.02	0.058 ± 0.005	0.19 ± 0.01	0.086 ± 0.006
Charge (mV)	2.01 ± 1.02	5.24 ± 0.28	−17.0 ± 0.62	−20.1 ± 1.84
EE%	-	65.9 ± 1.63	-	65.9 ± 1.63
LE%	-	1.64 ± 0.74	-	1.64 ± 0.74

**Table 2:** The IC_50_ values for BSA NPs, CLA, and CLA-BSA NPs against MCF7, MDA-231, A549, and HDF cell lines.

IC_50_ Against Cancer Cell Lines				
Treatments	MCF7	MDA-MB-231	A549	HDF
BSA NPs	>100 μM	>100 μM	>100 μM	>100 μM
CLA	>100 μM	>100 μM	66.1 ± 11.3	>100 μM
CLA-BSA NPs	>100 μM	>100 μM	47.5 ± 9.6	>100 μM

**Table 3:** The MIC values of three bacterial strains (*Bacillus cereus*, *Staphylococcus aureus*, *Streptococcus pyogenes*), treated with free CLA and CLA-BSA nanoparticles.

Tested Bacterial Strain	Treatment	MIC (µg/mL)
*Bacillus cereus*	CLA	0.053
	CLA-BSA nanoparticles	0.032
*Staphylococcus aureus*	CLA	0.05
	CLA-BSA nanoparticles	0.12
*Streptococcus pyogenes*	CLA	>32
	CLA-BSA nanoparticles	>32

## Data Availability

All data generated or analyzed during this study are included in this published article.

## Supplementary Materials

The following supporting information can be downloaded at https://www.mdpi.com/article/10.3390/pharmaceutics17060729/s1, Figure S1: HPLC chromotagram of CLA standard.
