# Supplementary figures and images for: Clarithromycin-Loaded Albumin-Based Nanoparticles for Improved Antibacterial and Anticancer Performance
Source: Pharmaceutics. 2025 May 31;17(6):729. doi: 10.3390/pharmaceutics17060729 (PMC12196031; doi:10.3390/pharmaceutics17060729)

## Supplementary

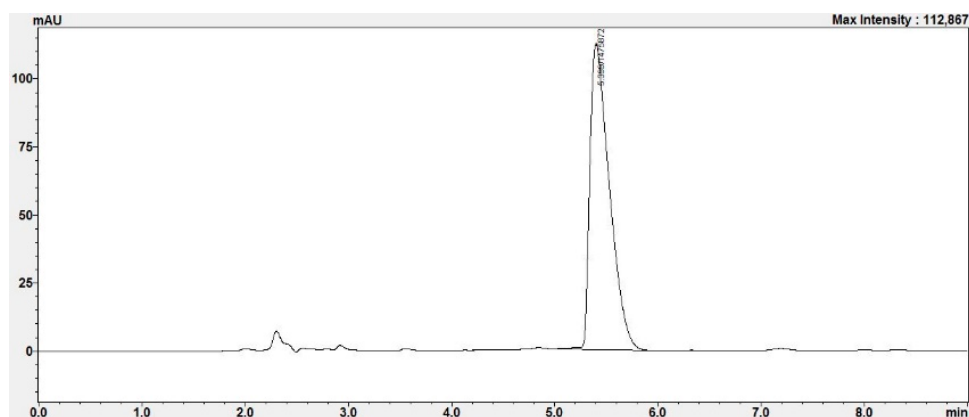

Figure S1. HPLC chromatogram of CLA standard.

Supplement: Supplementary file 1 [file pharmaceutics-17-00729-s001.zip › pharmaceutics-3611808-supplementary.pdf]
